# The influences of the environment and information on the complications of diabetes on patient outcomes

**DOI:** 10.1186/s12955-021-01798-6

**Published:** 2021-06-08

**Authors:** Kazuoki Inoue, Jun Watanabe, Eiichi Kakehi

**Affiliations:** 1 Department of Community-Based Family Medicine, Faculty of Medicine, School of Medicine, Tottori University, 86 Nishi-cho, Yonago-City, Tottori 683-8503 Japan; 2 Division of Community and Family Medicine, Jichi Medical University, 3311-1 Shimotsuke-City, Tochigi, 329-0498 Japan; 3 Tottori Municipal Hospital, 1-1 Matoba, Tottori-City, Tottori 680-8501 Japan

**Keywords:** Diabetes mellitus, Occupation-based, Problem-solving, Quality of life

## Abstract

This letter was written to address two concerns about the results of the paper published by Zeynep et al. (BMC Health Qual Life Outcomes 18:265, 2020). First, the differences between the two groups in the environment with or without occupation may strengthen the primary outcome results. Second, lack of information on the complications and treatments of diabetes makes interpretation of the results difficult.

Dear Editor,

We read with great interest this study, which clarified the effects of problem-solving therapy (PST) on the occupational performance, self-efficacy, and well-being of type 2 diabetic patients in Turkey [1]. However, we have two concerns about the methodology of this study.

First, the difference between the two groups in terms of whether they were in an environment with or without occupation might have significantly affected the results, so the results should be subtracted. In this study, although the participants were randomly assigned at Baseline, more participants in the intervention group than in the control group were engaged in work. Therefore, the Canadian Occupational Performance Measure (COPM) for primary outcomes is more likely to have a stronger effect on the amount of change because it affects the client’s environment [2]. The COPM is a questionnaire that asks about self-perceptions of occupational performance. Participants in the environment with occupation might find it easier to visualize and practice “problem definition,” “generation of alternatives,” “decision-making,” “solution implementation and verification,” in PST.

Second, this study does not describe the complications and treatment of diabetes, which we would like to know about. Hypoglycemia, a complication of diabetes, is said to reduce the work productivity of diabetic patients [3]. In addition, patients with type 2 diabetes are limited by the social and emotional aspects of insulin therapy [4]; they refuse insulin therapy because they feel that starting insulin will stigmatize them socially [5]. Thus, depending on the complications and treatment of diabetes, the results of this study may be weakened.

## Data Availability

Date sharing is not applicable to this article as no datasets were generated or analyzed during the present study.

## Abbreviations

PST: Problem-solving therapy
COPM: The Canadian Occupational Performance Measure
